# Modulation of T Lymphocyte Calcium Influx Patterns Via the Inhibition of Kv1.3 and Ikca1 Potassium Channels in Autoimmune Disorders

**DOI:** 10.3389/fimmu.2013.00234

**Published:** 2013-08-06

**Authors:** Csaba Orbán, Enikõ Biró, Enikõ Grozdics, Anna Bajnok, Gergely Toldi

**Affiliations:** ^1^First Department of Pediatrics, Semmelweis University, Budapest, Hungary

Currently available immunotherapies have improved the treatment of autoimmune diseases; however, these therapies are known to have considerable side-effects, such as increasing the susceptibility to infections. Therefore, there is an unmet need for novel immunosuppressive strategies with different mechanisms of action and higher specificity for disease-causing autoreactive T lymphocytes from existing immunomodulators.

The increase of the cytoplasmic calcium concentration from intra- and extracellular sources (i.e., the endoplasmic reticulum and store-operated calcium entry through the plasma membrane) is the cornerstone of T lymphocyte activation and functionality. In the course of lymphocyte activation, potassium channels maintain the driving force for sustained calcium influx from the extracellular milieu as they grant the efflux of potassium from the cytoplasm, thus conserving an electrochemical potential gradient between the intra- and extracellular spaces. There are two major types of potassium channels in T cells: the voltage-gated Kv1.3 and the calcium-activated IKCa1 channels. The relation between the calcium currents through calcium release-activated calcium (CRAC) channels and the efflux of potassium makes the proliferation and activation of lymphocytes sensitive to pharmacological modulation of Kv1.3 and IKCa1 channels, and provides an opportunity for targeted intervention. Specific inhibition of these channels results in a diminished calcium influx in lymphocytes and a lower level of lymphocyte activation.

Previous data suggest that selective modulation of lymphocyte activation through specific inhibition of potassium channels may be a possible therapeutic approach for the treatment of autoimmune disease (1–6). Beeton et al. showed that terminally differentiated effector memory T (TEM) cells play a pivotal role in the pathogenesis of autoimmunity (4). Wulff et al. described that the characteristic potassium channel phenotype of TEM cells in multiple sclerosis (MS) is Kv1.3high IKCa1low, contrasting naïve, and central memory T (TCM) cells, which exhibit a Kv1.3low IKCa1high channel phenotype (1). Therefore the therapeutic relevance of specific Kv1.3 channel inhibitors is of outstanding interest, as they may offer the possibility for selective modulation of pathogenic TEM cells, while naïve and TCM cells (needed for physiological immune responses) would escape the inhibition through upregulation of IKCa1 channel expression. Beeton et al. demonstrated that the symptoms of experimental autoimmune encephalitis, a murine model of MS, significantly improved after treatment with selective Kv1.3 inhibitors (5).

Although results from animal models are promising, limited data is available on the effects of potassium channel inhibition on T cell function in humans. Furthermore, besides naïve and memory T cells, alterations in the activation pattern of effector (CD4+ helper and CD8+ cytotoxic) T lymphocytes have not been described upon Kv1.3 and IKCa1 inhibition. Although these cells might have a less-specific role in the maintenance of autoreactivity compared to TEM cells, their inhibition have important consequences on the overall immune response. Therefore, over the recent years, we have investigated calcium influx characteristics in effector T cell subsets in a number of autoimmune disorders.

We isolated peripheral blood mononuclear cells from MS, rheumatoid arthritis (RA) and type 1 diabetes mellitus (T1DM) patients and applied a novel flow cytometry-based approach for the detection of calcium influx (7–10). Until the recent past, single-cell techniques were used for the investigation of calcium influx during lymphocyte activation. There has been no high-throughput method available to study the kinetics of lymphocyte activation in more subsets at the same time. Single-cell techniques are restricted by not being capable of characterizing this process in complex cellular systems, thus ignoring the interaction between the different lymphocyte subsets that may modulate the course of their activation. Therefore, over the recent years we have developed a novel algorithm that allows simultaneous monitoring of calcium influx in several lymphocyte subsets. Our software (FacsKin) fits functions to median values of the data of interest and calculates relevant parameters describing each function. By selecting the best fitting function, this approach provides an opportunity for the mathematical analysis and statistical comparison of kinetic flow cytometry measurements of distinct samples (more details available at www.facskin.com). Our findings indicate important differences in calcium influx kinetics in the studied autoimmune disorders compared to healthy controls.

## Multiple Sclerosis

Multiple sclerosis is an autoimmune disease affecting the central nervous system (CNS). The autoimmune reaction primarily destroys the myelin sheath covering the nerve cells. T lymphocytes play a key role in the pathogenesis of MS. They regulate the ongoing inflammatory process of the CNS which leads to the damage of the myelin sheath and axons. However, only a small part of T lymphocytes are myelin-specific autoreactive cells. Besides the demyelinating action of these cells of the CNS, the activation of peripheral lymphocytes also contributes to the pathogenesis and disease progression (11). In our investigations we measured samples of healthy individuals and MS patients with no immunomodulatory therapy, as well as MS patients treated with interferon beta (IFN-b), currently regarded as the most effective therapy of MS. We discovered increased sensitivity of CD8 cells to Kv1.3 channel inhibition in MS. Therefore, from the CD4-CD8 point of view, we demonstrated specific immunomodulation that may be beneficial in the therapy of MS via the selective suppression of CD8 cytotoxic lymphocytes over CD4 helper cells. However, this specificity is not present within the CD4 subset, since our results suggest that Th1 and Th2 cells are similarly suppressed upon the inhibition of Kv1.3 channels. Since the cytokine balance is of utmost importance in the regulation of the autoimmune reaction, the inhibition of the Th2 subset would probably result in a setback of therapeutic efforts in MS. Our findings are relevant in the light of observations regarding the contribution of TEM cells to the development of MS, as described above. Although the Kv1.3high IKCa1low pattern is found in both CD4+ and CD8+ TEM cells, we can assume that the majority of TEM cells are CD8+, since TEM cells express immediate effector function. This might provide an explanation for the increased sensitivity of CD8 cells to Kv1.3 channel inhibition in MS in our study.

We have also demonstrated important differences in calcium influx kinetics and lymphocyte potassium channel function in MS patients with and without IFN-b therapy. Selective blockers of the Kv1.3 channel might be promising drugs in combination therapy, supplementing the presently used IFN-b treatment. Our results indicate that IFN-b therapy is related to compensatory changes only in the Th1 subset in MS regarding calcium influx kinetics and the function of potassium channels. However, the increased function of the Th2 subset, and therefore the production of anti-inflammatory cytokines are less affected. This might contribute to a more effective treatment of the autoimmune process in this disorder (9).

## Rheumatoid Arthritis

The short-term activation of peripheral blood and synovial fluid T lymphocytes, especially that of autoreactive T cells plays a crucial role in initiating and maintaining the chronic inflammation in the joints of patients suffering from RA. These cells regulate the inflammatory process resulting in the destruction of the articular cartilage and also play a role in extra-articular damage. We investigated T lymphocyte calcium influx kinetics following activation in peripheral blood of recently diagnosed RA patients compared to healthy individuals. Our results indicate that margatoxin (MGTX), a specific blocker of the Kv1.3 channel acts differentially on calcium influx kinetics in major peripheral blood effector lymphocyte subsets of RA patients. The immunomodulatory effect of Kv1.3 channel inhibition is predominantly seen in cytotoxic (CD8) T cells in RA. However, this effect does not seem to be as specific as reported before by Beeton and colleagues in case of TEM cells (2), since anti-inflammatory Th2 cells are also affected to a noteworthy extent. This subset has an important role in counterbalancing the ongoing inflammatory process, and therefore its inhibition is not useful in the treatment of RA. A reason for limited specificity of Kv1.3 inhibition in the peripheral lymphocytes might be the differential distribution of disease-associated autoreactive T cells in RA patients on local and systemic levels. In the synovial fluid (locally), autoreactive TEM cells, expressing high numbers of Kv1.3 channels are abundantly present. However, this Kv1.3 pattern was not detected in peripheral blood T cells, because autoreactive TEM cells are infrequent in the circulation. Peripheral blood T cells were predominantly found to be naive and TCM cells (2).

## Type 1 Diabetes Mellitus

Our results indicate that the sensitivity of T1DM lymphocytes to the inhibition of Kv1.3 channels is increased compared to healthy individuals. It has been hypothesized that beneficial effects of Kv1.3 channel inhibition by MGTX are due to the dominance of Kv1.3 channels on activated TEM cells (1, 2). It was also presumed that MGTX would inhibit the activation of CD8 lymphocytes responsible for cytotoxic destruction of pancreatic beta cells. Nevertheless, our findings support that Kv1.3 channels have an important role in each investigated lymphocyte subset in T1DM, including Th2 lymphocytes acting as counterbalancing factors in the development of T1DM through the production of anti-inflammatory cytokines (12). Therefore, administration of Kv1.3 channel inhibitors would not have an exclusive effect on cells responsible for the autoimmune response in T1DM, but may have an impact on the activation characteristics of immune cells in general (8).

## Summary

Based on our results, a number of dominant features were identified that were present in the investigated autoimmune diseases. First, the time when the peak of calcium influx was reached decreased in autoimmune patients compared to healthy individuals, indicating that these cells are in a state of sustained reactivity due to the ongoing autoimmune reaction.

Earlier studies were limited to the investigation of potassium channels in naive and memory lymphocytes. We have extended these findings to significant effector T lymphocyte subsets, and found a different pattern of sensitivity to the inhibition of lymphocyte potassium channels in Th1 cells of autoimmune patients compared to healthy individuals. In healthy controls the inhibition of the IKCa1 channel decreased calcium influx in Th2 and CD4 cells to a lower extent than in Th1 and CD8 cells. On the contrary, the inhibition of Kv1.3 channels resulted in a larger decrease of calcium entry in Th2 and CD4 than in Th1 and CD8 cells. In the investigated autoimmune patients a greater decrease of calcium influx upon the inhibition of the Kv1.3 channel than that of the IKCa1 channel was observed in Th1 cells. This finding is of special interest, since Th1 cells are regarded as key players in the mediation of pro-inflammatory responses.

However, the selectivity of the investigated inhibitors was limited in our experiments, as they did not only affect a single subset, as previously suggested. Although in earlier observations the inhibition of Kv1.3 channels specifically blocked the function of TEM cells, our investigations extending to significant effector T lymphocyte subsets demonstrated that the inhibitory effect is present not only in disease-associated CD8 and Th1 cells, but also in the anti-inflammatory Th2 subset. The induced decrease in their function could lead to unwanted side-effects and in a setback of therapy *in vivo*.

Therefore, further studies, including the analysis of functional consequences (such as cytokine production or proliferation) of lymphocyte potassium channel inhibition are needed on human samples and experimental models to judge the usefulness of this approach in the fight against autoreactive lymphocyte subsets and harmful cellular responses in autoimmune patients.
